# Shared decision-making and specific informed consent in patients with aortic aneurysms

**DOI:** 10.31744/einstein_journal/2023AO0197

**Published:** 2023-08-03

**Authors:** Marcela Juliano Silva Cunha, Marcelo Passos Teivelis, Cynthia de Almeida Mendes, Conrado Dias Pacheco Annicchino Baptistella, Pedro Vasconcelos Henry Sant´Anna, Nelson Wolosker

**Affiliations:** 1 Hospital Municipal da Vila Santa Catarina Dr. Gilson de Cássia Marques de Carvalho Brazil Hospital Municipal da Vila Santa Catarina Dr. Gilson de Cássia Marques de Carvalho; Hospital Israelita Albert Einstein, São Paulo, SP, Brazil.; Hospital Israelita Albert Einstein São Paulo SP Brazil; 2 Hospital Israelita Albert Einstein São Paulo SP Brazil Hospital Israelita Albert Einstein, São Paulo, SP, Brazil.; 3 Faculdade Israelita de Ciências da Saúde Albert Einstein Hospital Israelita Albert Einstein São Paulo SP Brazil Faculdade Israelita de Ciências da Saúde Albert Einstein, Hospital Israelita Albert Einstein, São Paulo, SP, Brazil.

**Keywords:** Aortic aneurysm, Decision making, shared, Patient rights, Consent forms, Vascular surgical procedures

## Abstract

**Objective:**

To analyze the refusal rate of elective aortic aneurysm surgery in asymptomatic patients after the presentation of a detailed informed consent form followed by a meeting where patient and their families can analyze each item.

**Methods:**

We conducted a retrospective analysis of 49 patients who had aneurysms and were offered surgical treatment between June 2017 and February 2019. The patients were divided into two groups: the Rejected Surgery Group, which was composed of patients who refused the proposed surgical treatment, and the Accepted Surgery Group, comprising patients who accepted the proposed surgeries and subsequently underwent them.

**Results:**

Of the 49 patients, 13 (26.5%) refused surgery after reading the informed consent and attending the comprehensive meeting. We observed that patients who refused surgery had statistically smaller aneurysms than those who accepted surgery (9% *versus* 26%). These smaller aneurysms were above the indication size, according to the literature.

**Conclusion:**

One-quarter of patients who were indicated for elective surgical repair of aortic aneurysms rejected surgery after shared decision-making, which involved presenting patients with an informed consent form followed by a clarification meeting for them and their families to analyze each item. The only factor that significantly influenced a rejection of the procedure was the size of the aneurysm; patients who rejected surgery had smaller aneurysms than those who accepted surgery.

## Highlights

Up to 26% of patients with aortic aneurysms refused surgical repair.

The proposed technique, whether open or endovascular, did not influence patients’ decisions.

Patients with smaller aneurysms were more likely to refuse aortic aneurysm treatment.

**Figure:**
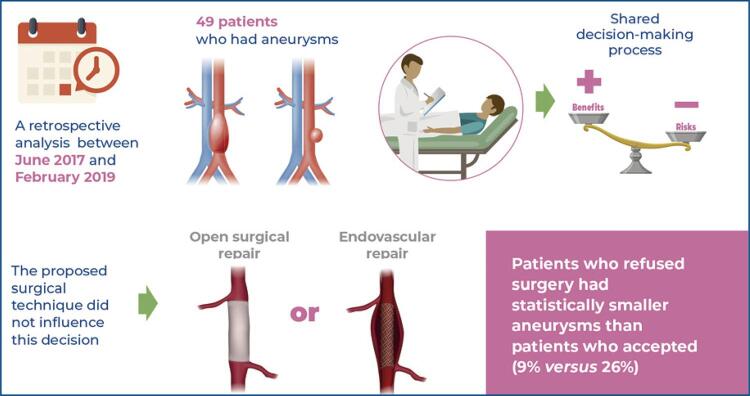

## INTRODUCTION

The shared decision-making (SDM) process is a two-way communication in which the patient and surgeon collaborate in choosing the appropriate treatment, incorporating the patient’s preferences and values as well as the best scientific evidence. Shared decision-making is considered a tool of high ethical and moral standards, and it is important not only because it gives the patient the choice of whether to be treated for their disease but also because it raises awareness of the different risks associated with each procedure and sets expectations for each choice.^(1)^

Studies have shown that patients undergoing vascular surgery have the desire to participate more actively in their treatment; however, they find it difficult to express themselves. They prefer to know all available therapeutic options, not only those that the surgeon considers appropriate.^(2)^ With this knowledge, patients can choose the most appropriate therapeutic modality for themselves (including non-operative approaches), making them responsible for the therapeutic decision.

In Brazil and most of the world, informed consent (IC) for elective surgeries is usually general and standardized for all types of surgery, with some blank spaces to be filled in by the physician based on the variability of each case. Surgeons do not typically apply a specific form to the patient’s disease. The generalized risks, such as bleeding and infection, as well as the risks associated with blood transfusion, are usually described for elective procedures. However, with these documents, patients cannot distinguish between therapeutic alternatives, including non-operative treatment.

At our institution, we routinely offer a personalized and detailed document, as part of the SDM process, explaining the patients’ disease in case of elective vascular surgeries, the proposed surgeries, and all risks and benefits. This document includes a section that must be handwritten by the patients or their families to demonstrate their understanding of the information. These data are only filled in after a comprehensive meeting is held between the doctor, patients, and their families, which can take up to an hour.

## OBJECTIVE

This study aimed to analyze the refusal rate for elective aortic aneurysm surgery in asymptomatic patients after the presentation of a detailed informed consent form, followed by a meeting where the patients and their families analyze each item. Additionally, we aimed to identify specific factors of refusal by comparing the epidemiological data, aneurysm characteristics, and surgical techniques proposed among patients who accepted to undergo the procedures and those who rejected them.

## METHODS

We conducted a retrospective study of the medical records of 49 patients diagnosed with asymptomatic aorto-iliac aneurysms who were followed by the vascular surgery team of the *“Hospital Municipal da Vila Santa Catarina Dr. Gilson de Cássia Marques de Carvalho”* between June 2017 and February 2019. This study was approved by the Hospital’s Ethics Committee of the *Hospital Israelita Albert Einstein* (CAAE: 13181919.3.0000.0071; # 3.354.399). Ethical approval was given by the Institutional Review Board, and informed consent was obtained from the included patients. All patients were considered fit for surgery after evaluation by the surgical team and the clinical team that managed the preoperative evaluation.

The IC described the risk of death, paraplegia and spinal cord ischemia, stroke, the risk for colostomy, re-operation due to hernia, limb ischemia/limb amputation, the risk for hemodialysis, and the higher risk of re-intervention for endovascular cases. In addition, the IC provides clear and individualized information concerning the proposed treatment for each patient and the specific related risks.

All patients took home the IC forms, and after sharing them with their families, they returned for a comprehensive meeting between the doctor, family, and patient. During the meeting, the document was read in its entirety, and all points were discussed and clarified. Blank spaces were provided for the patients or their families to write down any doubts they may have and to indicate their understanding of the proposed surgery and its related risks. Six physicians (including two experienced vascular surgeons and four senior residents) participated in these meetings.

The clinical and epidemiological information of patients were collected (age, sex, diagnosis, percentage by which the aneurysm exceeded the size that would have a formal indication for surgery, proposed/used surgical technique, presence of hypertension, diabetes mellitus, coronary disease, cerebrovascular disease, history of smoking, chronic renal disease and body mass index) in addition to the disease for which the surgical treatment was offered and the proposed surgical technique when more than one technique was available.

The indication for surgery in all patients was based on the guidelines of the Society of Vascular Surgery and European Society of Vascular Surgery, and the following diameters were considered as thresholds for different types of aneurysms: abdominal aortic aneurysms of 5.5cm in men and 5.0cm in women; thoracic aortic aneurysms diameters of 6cm; thoracoabdominal aneurysms diameters of 6cm; and iliac artery aneurysm diameters (common, internal or external) of 3.0cm.^(3,4)^The percentage above the threshold (PaT) was calculated for each patient, which represents the percentage of the aneurysm size that exceeded the minimum diameter in the formal surgical indication. To calculate the PaT, the diameter of the artery with the aneurysm that exceeded the threshold value was divided by the threshold value of the surgical indication for that specific artery and multiplied by 100. The decision between open and endovascular techniques considered the materials available in our public service and always respected the instructions for use.

The patients were divided into two groups: the Rejected Surgery Group, which was composed of patients who refused the proposed surgical treatment, and the Accepted Surgery Group, which comprised patients who accepted the proposed surgeries and subsequently underwent them.

Initially, the demographic characteristics between the two groups were analyzed, and then the relationship between surgery rejection and PaT was evaluated.

Qualitative characteristics were described using absolute and relative frequencies, and quantitative characteristics were described using summary measurements (mean, standard deviation, median, minimum and maximum). Fisher’s exact tests were used to verify the associations of the qualitative characteristic of the refusal of surgery, and quantitative characteristics were compared using student’s t-tests. The analyses were performed using SPSS for Windows version 22.0 software (SPSS Inc., Chicago, IL), and the tests were performed with a significance level of 5%.

## RESULTS

Of the 49 patients, 13 (26.5%) refused surgery after reading the IC form and after attending the described meeting. These patients were referred to primary care with instructions to return to our service if they changed their decision or had any new doubts. Unexpectedly, the type of surgery proposed, open or endovascular, did not seem to directly influence this decision. We did not offer the endovascular approach to patients who refused open repair since one reason for primarily offering open repair to some patients was the aortic anatomy and the non-conformance to the instructions for use.

The demographic data and data regarding the type of surgery recommended for both groups are presented in table 1. We observed that there was no significant difference between the groups, and most of the patients were male, smokers, and hypertensive. Only 2% of the cases were isolated thoracic aneurysms, 4% were thoracoabdominal aneurysms, 15% were trans renal aneurysms, and 51% were infrarenal abdominal aortic aneurysms. The type of aortic aneurysm did not significantly influence patients’ decisions. Regarding the proposed surgical technique, 61.5% of the patients who did not accept surgical treatment received the proposal of an open repair. Among the patients who chose to undergo surgery, 55.5% underwent open repair, and there were no statistically significant differences.

**Table 1:** Qualitative and quantitative analyses of each group

	Rejected Surgery Group	Accepted Surgery Group	Total	p value
	
	n=13	n=36		
Age				
Mean±SD	70.69±6.44	72.92±6.85		0.298^†^
BMI				
Mean±SD	26.41±3.76	25.73±4.73		0.604^†^
Sex, (%)				
Female	4 (30.8)	13 (36.1)	17	>0.999*
Male	9 (69.2)	23 (63.9)	32	
Hypertension, (%)				
Yes	10 (76.9)	30 (83.3)	40	0.683*
Diabetes, (%)				
Yes	2 (15.4)	12 (33.3)	14	0.297*
Myocardial infarction, (%)				
Yes	3 (23.1)	7 (19.4)	10	>0.999*
Stroke/transient cerebral ischemia, (%)				
Yes	2 (15.4)	4 (11.1)	6	0.65*
Smoking, (%)				
Yes	11 (84.6)	31 (86.1)	42	>0.999*
COPD, (%)				
Yes	2 (15.4)	10 (27.8)	12	0.474*
CKD				
Yes	2 (15.4)	9 (25.0)	11	0.703*
Surgical technique, (%)				
Open	8 (61.5)	20 (55.6)	28	0.755*
Endovascular	5 (38.5)	16 (44.4)	21	
Total	13 (26.5)	36 (73.5)	49	

* Fisher’s exact test; ^†^ Student’s *t*-test.

COPD: chronic obstructive pulmonary disease; CKD: chronic kidney disease.

The qualitative and quantitative analyses for each group are described in table 1. We observed that patients who refused surgery had significantly smaller aneurysms than those who accepted surgery (9% above the threshold *versus* 26% above the threshold) (Table 2). Therefore, the only factor that significantly influenced patients’ decisions to accept or refuse the proposed surgery was the size of the aneurysm, implying that the smaller the aneurysm, the higher the rate of surgical refusal.

**Table 2:** Percentage above the threshold in patients who refused and those who accepted surgery

Refusal	Mean±SD	Median	Minimum	Maximum	n	p value
No	0.26±0.24	0.17	0	0,94	36	0.003*
Yes	0.09±0.11	0.05	0	0,32	13	

* Student’s *t*-test.

## DISCUSSION

Vascular surgeons can describe the risk of aneurysm to patients. Aneurysms can be portrayed as having minimal risk, neglected, or may be described as ticking time bombs. A Dutch study from 2015 interviewed 10 men with aneurysms ranging from 35 to 49mm in diameter. Most of the patients were able to explain the basic concept of aneurysms; however, several had conceptual doubts. The authors found that the patients felt comfortable with the surveillance of small aneurysms; nonetheless, they observed that at size of 55mm patients perceived aneurysms as dangerous.^(5)^ The study noted that most patients had a low level of education, although this low level was not defined. All patients trusted their vascular surgeon to know what was best for them and considered them the most important source of information.^(5)^

Similar to Tomee et al.,^(5)^ in our clinical practice, we had the perception that patients with relatively smaller aneurysms were more likely to refuse surgery owing to a greater fear of complications. We did not find any studies that analyzed the rates of refusal for surgical treatment in patients with aortic aneurysms after the introduction of instruments that would assist with the SDM or after the implementation of a detailed IC form; hence, we were motivated to analyze the profile of patients who refused aortic surgery in our service.

Currently, some patients with aneurysms are inconsistently informed about their disease and the available treatments. The amount of information provided is less than that which is legally required. Informing the patient about the available treatment options is an ethical and moral issue. The approach to raising awareness may not involve technological assistance, such as letting the patient watch or read informative materials about the disease using machines, computerized videos, or other impersonal ways. It is preferable to use human resources since there is a professional available at the doctor’s office to give personalized and sensitive information to these patients.^(6)^ Our perception is that aneurysms whose size was closer to the size threshold (9% or less) did not arouse as much risk perception in patients. This size of 9% above the threshold was statistically obtained using the Student’s *t*-test.

From our perspective, the reason why patients with aneurysms refuse to undergo surgical repair is related to their understanding of the information presented and discussed by our team. Asymptomatic patients consider the risk of death small when they are not operated on and accept this risk better than possible surgical complications, which for them would probably have a severe impact on their quality of life. For them, the aneurysm was not exactly a ticking time bomb.

Shared decision-making prevents excessive treatment and potentially reduces costs or at least allows resources to be spent only on patients who truly desire operative treatment. In addition to the costs of the procedure itself (in our country, the endovascular technique is more expensive), there are costs associated with follow-ups, examinations, and re-operations. Aneurysm repair increases health care costs, whether public or private.^(7)^On the other hand, some patients who refuse surgery may experience aneurysm rupture, and emergency treatment in such cases can be more expensive than elective treatment. In addition to clarifying the patient’s disease and proposed treatment, the IC form also protects both patients and doctors, ethically and legally.

Patients with lower education levels, more anxiety, and a worse prognosis tend to prefer less patient-centered care.^(8)^ The national demographic data shows that only 6.6% of Brazilian public health users have higher education levels, 31.1% have completed high school, 54.8% have only completed elementary school, and 7.5% are illiterate.^(9)^ One limitation of our study is that we did not evaluate the education level of the patients; however, there was an important language barrier in meetings with the study population. We believe that the information was better understood by the patients and their families in written form than when it was only verbally expressed, which might have influenced the increased refusal number.

This study has certain limitations. First, the number of patients was relatively small, and our findings may only apply to the Brazilian population, whose socioeconomic and educational characteristics may impact SDM. Additionally, the study was conducted in a public service hospital with limited resources, and complex endovascular techniques (such as fenestrated or branched prostheses or the use of endoanchors) were not financially viable options. Thus, patients with more complex anatomy were offered only the possibility of open repair, which possibly brings with it the perception of greater risks. Further, our study was not randomized; hence, we cannot affirm that this refusal rate would not be the same in patients without this specific IC.

## CONCLUSION

Our study found that one-quarter of patients who were recommended for elective repair of aortic aneurysms rejected surgery after shared decision-making, which involved presenting an informed consent form followed by a clarification meeting for the patients and their families to analyze each item. The only factor that significantly influenced a decision to reject the procedure was the size of the aneurysm; hence, patients who rejected surgery generally had smaller aneurysms than those who accepted surgery.
